# Caregiver-Associated Physical Activity Patterns, Dietary Behaviors and Interventional Beliefs in Individuals with Down Syndrome: Insights from a Large European Survey

**DOI:** 10.3390/nu18111692

**Published:** 2026-05-26

**Authors:** Thomas Cahill, Valerie Nalesso, Pat Clarke, Maria Martinez de Lagran, Andre Strydom, Li Chan, Marie-Claude Potier, Johannes Beckers, Klaus Langohr, Pietro Liò, Rafael de La Torre, Laura Forcano, Anne Hiance-Delahaye, Yann Hérault, Mara Dierssen, GO-DS21 Consortium

**Affiliations:** 1 Centre for Genomic Regulation (CRG), The Barcelona Institute of Science and Technology, Dr. Aiguader 88, 08003 Barcelona, Spain; thomas.cahill@crg.eu (T.C.); maria.martinez@crg.eu (M.M.d.L.); 2 Institut de Génétique et de Biologie Moléculaire et Cellulaire (IGBMC), INSERM, CNRS, Université de Strasbourg, UMR7104, U1258, 67404 Illkirch-Graffenstaden, France; nalesso@igbmc.fr; 3 European Down Syndrome Association, 1210 Brussels, Belgium; pat@patclarke.eu; 4 Institute of Psychiatry, Psychology, and Neuroscience, King’s College London, London SE5 8AB, UK; andre.strydom@kcl.ac.uk; 5 South London and Maudsley NHS Foundation Trust, London SE5 8AZ, UK; 6 The LonDowns Consortium, London SE5 8AF, UK; 7 Centre for Endocrinology, William Harvey Research Institute, Barts and the London School of Medicine and Dentistry, Queen Mary University of London, London EC1M 6BQ, UK; hhw284@qmul.ac.uk; 8 Institut du Cerveau—Paris Brain Institute-ICM, CNRS, APHP, Hôpital de La Pitié Salpêtrière, Inserm, Sorbonne Université, 75013 Paris, France; marie-claude.potier@icm-institute.org; 9 Institute of Experimental Genetics, German Research Centre for Environmental Health, Helmholtz Zentrum München, Ingolstaedter Landstr. 1, 85764 Neuherberg, Germany; johannes.beckers@helmholtz-munich.de; 10 German Centre for Diabetes Research (DZD), Ingolstaedter Landstr. 1, 85764 Neuherberg, Germany; 11 Experimental Genetics, School of Life Sciences Weihenstephan, Technische Universität München, Altekademie 8, 85354 Freising, Germany; 12 Integrative Pharmacology and Systems Neuroscience Research Group, Neuroscience Research Program, Hospital del Mar Research Institute, 08003 Barcelona, Spain; klaus.langohr@upc.edu (K.L.); lforcano@researchmar.net (L.F.); 13 Department of Statistics and Operations Research, Universitat Politècnica de Catalunya—BarcelonaTECH, 08034 Barcelona, Spain; 14 Department of Computer Science and Technology, University of Cambridge, Cambridge CB3 0FD, UK; pl219@cam.ac.uk; 15 CIBER de Fisiopatología de la Obesidad y Nutrición, Instituto de Salud Carlos III, 28029 Madrid, Spain; 16 Institut Jérôme Lejeune, 75015 Paris, France; anne.hiance-delahaye@institutlejeune.org; 17 PHEN—Institut Clinique de la Souris (PHEN-ICS), INSERM, CNRS, Université de Strasbourg, UAR2062, US66, 67404 Illkirch-Graffenstaden, France; 18 CNRS, CELPHEDIA-Core, UAR2052, 94800 Villejuif, France; 19 Universitat Pompeu Fabra (UPF), Campus Uuniversitari Mar, 08005 Barcelona, Spain; 20 Biomedical Research Networking Center for Rare Diseases (CIBERER), 08003 Barcelona, Spain

**Keywords:** Down syndrome, caregiver confidence, interventions, physical activity, diet, obesity

## Abstract

Background: Lifestyle factors such as diet and physical activity significantly impact on the risk of obesity in individuals with Down syndrome (DS). However, in the absence of national nutritional guidelines in individuals with DS, further work is needed to understand their dietary and physical activity patterns. In this work we retrieved caregivers’ responses on those aspects. Methods: We analyzed data from a cross-sectional online survey of caregivers of individuals with DS conducted as part of the GO-DS21 project and reported in the accompanying paper (nutrients-4216283) (n = 764). We explored physical activity patterns, dietary habits, beliefs around weight-loss interventions and caregiver confidence that family members with DS would engage in a healthier lifestyle. Associations were examined using correlation analysis, and cumulative and binary logistic regression models. Results: Caregivers reported that most individuals with DS exercised 1–3 times per week, with frequency declining with age. Males were more likely to exercise daily than females. Caregiver exercise frequency was positively correlated with that of their DS family member (ρ = 0.521, *p* < 0.001), suggesting clustering of shared health behaviors within households. In adjusted models, caregivers who exercised regularly had up to thirteen-fold higher odds of having a physically active family member with DS (aOR = 13.02, 95% CI: 7.40–24.06, *p* < 0.001). Fried food consumption and higher snack frequency were independently associated with perceived obesity status, while sugar-sweetened beverage consumption was not. Caregivers favored exercise as a weight-loss strategy, while anti-obesity drugs were endorsed by only 11% of caregivers primarily and were more likely to be endorsed when obesity was perceived (aOR = 4.21, 95% CI: 2.44–7.39, *p* < 0.001). Finally, caregiver confidence that their family member with DS would engage in healthier behaviors was associated with perceived obesity status and strongly associated with higher physical activity levels (aOR 14.68, 95% CI: 6.59–33.40, *p* < 0.001). Conclusions: In this large European caregiver survey, reported consumption of selected energy-dense foods was generally low, although fried food intake and higher snack frequency were associated with perceived obesity. Physical activity patterns were closely aligned between caregivers and individuals with DS, suggesting shared household health behaviors. These findings highlight the importance of involving caregivers and family environments in lifestyle interventions aimed at supporting physical activity and weight management in individuals with DS.

## 1. Introduction

Obesity, characterized by a BMI > 30, has been associated with an increased risk of noncommunicable diseases (NCDs), including cardiovascular diseases, diabetes, certain cancers, neurological disorders, respiratory diseases, and digestive disorders [1]. Individuals with Down syndrome (DS) are at increased risk of obesity compared with the general population due to a combination of physiological and behavioral factors including lower resting energy expenditure, metabolic dysregulation, poorer diet habits, and lower physical activity (PA) levels [2]. Understanding modifiable lifestyle factors is therefore particularly important in this population.

Individuals with DS are consistently less physically active than those without the condition [3], likely due to DS-specific physiological and environmental factors including hypotonia, motor coordination difficulties, reduced energy expenditure [4], reduced lean muscle mass [5], and mitochondrial dysfunction [6], as well as limited access to adapted PA programs. PA is important not only for weight management, but also for muscle strength, balance, autonomy, social participation, self-esteem, confidence, and cognitive health. Despite these benefits, there are no DS-specific PA guidelines, and most individuals with DS do not meet recommended levels of moderate-to-vigorous exercise across age groups [7,8]. Evidence also suggests that females with DS are less physically active than males [7].

Dietary habits are also important determinants of obesity risk and overall health. Individuals with DS may face specific challenges in maintaining a healthy diet, including selective eating, related to sensory sensitivity and oral-motor difficulties affecting chewing and swallowing. Previous studies suggest a higher consumption of energy-dense foods, such as sweets and processed foods, and lower intake of fruits, whole grains, and dairy products [9,10,11]. However, there are no universally adopted DS-specific nutritional guidelines, and recommendations generally mirror those for the general population [11]. Recent GO-DS21 clinical recommendations emphasize that overweight and obesity management in DS should adopt a holistic, individualized approach that combines tailored dietary guidance, physical activity promotion, management of co-occurring health conditions, and active involvement of families and peers [12]. Dietary patterns may also be shaped by caregiver practices and attitudes, yet evidence on how caregiver perceptions and beliefs influence diet and weight management in DS remains limited.

In recent years, weight-loss interventions, including anti-obesity drugs such as GLP-1 agonists, have shown substantial efficacy in the general population [13]. However, little is known about caregiver attitudes toward these interventions for family members with DS.

We hypothesized that caregiver practices and attitudes are associated with PA and dietary patterns in their family members with DS. This study therefore aimed to characterize PA patterns, dietary habits, caregiver confidence, and attitudes toward weight-loss interventions in a large European caregiver cohort. Together with the companion paper (nutrients-4216283), our findings provide a broader overview of obesity-related perceptions and lifestyle behaviors in individuals with DS.

## 2. Materials and Methods

### 2.1. Design and Participants

This study uses data from the same cross-sectional caregiver survey described in the accompanying companion paper (nutrients-4216283) and delivered as part of the GO-DS21 project. The questionnaire was developed within a Knowledge, Attitudes and Practices framework [14] and incorporated elements of the validated Obesity Awareness and Insight Scale (OASIS) [15], using Likert-scale items. The full instrument contained 30 questions that were grouped into KAP domains. Practice-related items captured the physical activity frequency of both individuals with DS (“How frequently does your family member with DS practice sport”: Never to Everyday) and caregivers (“How often do you exercise?”: Never to 6–7 days/weeks), as well as dietary behaviors including SSB consumption, fried food consumption, snack frequency, and structured meal patterns. Attitudinal responses included perceptions of obesity where caregivers reported obesity perceptions regarding their family member with DS (“I consider my family member to be obese”; Definitely/Probably/Don’t know/Probably not/Definitely not). It also captured caregiver beliefs regarding weight-loss interventions (anti-obesity drugs, meal replacers, fasting/skipping meals, and aerobic and anaerobic exercise) and caregiver confidence in seven health behaviors in their family member with DS (e.g., engaging in physical activity, avoiding fried food, walking to near-by places). The survey was disseminated through various European DS associations, including EDSA, Down España, and Trisomie 21 France in countries such as UK, Spain, France, and Germany, receiving 764 respondents. This cohort may therefore over-represent more engaged or health-aware caregivers and should be considered when generalizing findings. Eligible participants were adult caregivers with a family member with DS and no minimum age restriction applied to family members with DS with age ranges spanning from 1–15 to 16–30, 31–45, 46–50 and >51 years. Prior to accessing the questionnaire, participants received written information outlining the study objectives, the voluntary nature of participation and applicable data protection measures. This survey was fully anonymous and did not collect directly identifiable personal or health data; completion was therefore considered to constitute informed consent. The questionnaire adapts previously validated instruments rather than constituting a novel psychometric tool and formal psychometric validation was not performed which is acknowledged as a limitation of the study. While nutrients-4216283 focused on caregiver perceptions of obesity, awareness of associated risks, and healthcare engagement, the present analysis examines physical activity patterns, dietary behaviors, beliefs regarding weight-loss interventions, and caregiver confidence.

### 2.2. Statistical Analyses

All analysis was performed in R (v4.3.2) (R Core Team, 2025). The data processing and statistical analysis were conducted using the tidyverse, readxl, janitor, ordinal, broom, pscl, and car packages. Missingness was assessed using the naniar package and data was visualized using ggplot2, scales, patchwork and ggaluvial.

#### 2.2.1. Missingness and Sparsity

Missingness across all analysis variables was minimal (<1.7%). MAR tests using logistic regression of missingness indictors on sex and age group were not significant for DS PA (*p* ≥ 0.28) or most dietary variables supporting MCAR and complete case analysis. An association was observed for age >46 years and dietary missingness (*p* = 0.031); however, due to the small proportion of missingness, complete case analysis was justified. Sparsity was assessed via cell counts: cells of ≤5 responses were collapsed as described below.

#### 2.2.2. Data Preprocessing

A cleaned dataset was generated prior to analysis with standardized and recoded variables. Age was retained in two forms: Age_5 preserved all five age groups and was used for descriptive analysis, and Age_4 combined the two oldest age groups (46–50 and >51 years). Survey languages were recoded to Spanish, French, English and Germans and collapsed into Spain vs. Other Europe to ensure adequate sample size for regression analysis.

To explore the relationship between the caregiver and DS family member exercise patterns, we performed Spearman’s rank correlation by coding exercise frequencies into ascending ordinal scales. Caregivers were asked “How often do you exercise?” and “How frequently does your family member with DS practice sport?”. Caregiver responses included “Never”, “Once a week”, “2–3 days a week”, “4–5 days a week”, and “6–7 days a week”, while responses regarding DS family members included “Never”, “Once a month”, “1–3 times a week”, “4–6 times a week”, and “Everyday”. To streamline comparisons, responses were aligned to represent increasing levels of PA. Caregiver responses of “Once a week” were merged with “2–3 days a week” to form a single “1–3 days a week” category, matching the DS family member scale. While “4–5 days per week” was compared with “4–6 times a week”, and “6–7 days” a week with “Everyday”. DS responses of “Once a month” were excluded as there were no equivalent comparisons among caregiver responses.

DS PA frequency was ordered as a five-level ordered factor (DS-PA_5_: Never; Once a month; 1–3 times/week; 4–6 times/week; Everyday). This was used in the caregiver confidence model as an outcome. For the caregiver exercise model this was dichotomized into Inactive (Never or Once a month) and Active (>1–3 times/week) (DS-PA_2_). Caregiver exercise frequency was retained at five levels (CG-EX_5_: Never; Once a week; 2–3 days/week; 4–5 days/week; 6–7 days/week) and collapsed into three levels: (CG-EX_3_: Low = Never or Once a month; Moderate = 2–3 days/week; and High = 4–5 or 6–7 days/week). Caregiver confidence (CONF_5_: Not at all confident to Extremely confident) was retained as a five-level predictor.

Sugar-sweetened beverages (SSB) and fried food consumption were each collapsed into three levels (SSB_3_ and FF_3_: Low = Never/Rarely; Moderate = 1–2 times/week; or high ≥ 3 times/week). Snack frequency was collapsed into a binary scale (SNACK_2_: 0–1 vs. >2 per day) due to a structural zero in “2 snacks/day”. Perceived obesity was dichotomized into a binary outcome, Yes (“Definitely/Probably”) vs. No (“Definitely not/Probably not””), excluding “Don’t know” due to their ambiguity in a binary framework. Interventional beliefs were modeled with separate binary logistic models with the same dichotomization, and with perceived obesity as an additional covariate.

#### 2.2.3. Descriptive and Regression Analyses

The distribution of responses were examined by cross tabulation and visualized with stacked bar charts, heat maps, and alluvial diagrams.

Three classes or multivariable regression models were fitted and adjusted for age (Age_4), sex and region based on their known associations with PA and dietary patterns. A binary logistic regression (glm(), binomial) was used to assess the association between caregiver exercise frequency (CG-EX_3_) and DS PA levels (DS-PA_2_). A cumulative link model (clm(), ordinal package) was used to assess the association between caregiver confidence (CONF_5_) and DS PA (DS-PA_5_). Proportional odds assumptions were tested by predictor using nominal_test().

To explore interventional beliefs, separate binary logistic regression models were fitted with positive responses defined as “Definitely/Probably” and negative responses defined as “Definitely not/Probably not” for anti-obesity drugs fasting/skipping meals and meal replacers. Anaerobic and aerobic interventions were excluded from analysis due to ceiling effects.

Binary logistic regression analysis was used to examine whether SSB consumption (SSB_3_), fried food consumption (FF_3_), and snack frequency (SNACK_2_) predicted perceived obesity status, excluding “Don’t know” responses. Multi-collinearity was assessed using variance inflation factors (threshold > 5). Results are reported as adjusted odds ratios (aOR, with 95% confidence intervals (CI)). Model fit was assessed using McFaddens R^2^. Significance was set at α = 0.05.

## 3. Results

### 3.1. Practices: Shared Physical Activity Patterns Between Caregivers and Family Members with DS

**Figure 1:**
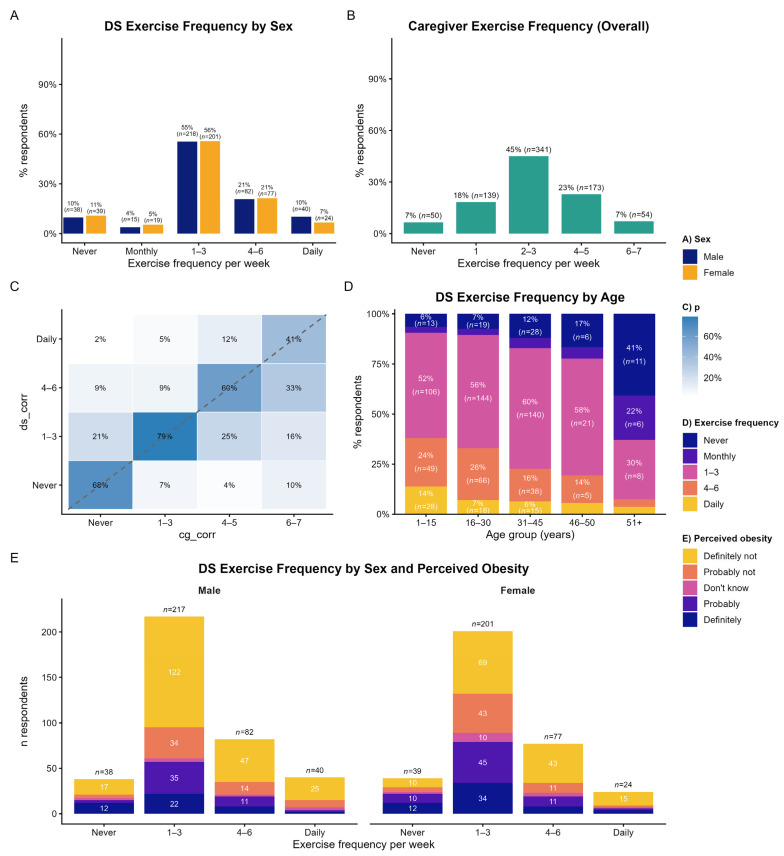
Physical activity patterns in individuals with Down syndrome and their caregivers. (**A**) Reported exercise frequency of individuals with DS stratified by sex. Most males and females were reported to exercise 1–3 times per week, with broadly similar distributions across sex. (**B**) Overall caregiver exercise frequency. Most caregivers reported exercising 2–3 days per week, followed by 4–5 days per week. (**C**) Relationship between caregiver and family member with DS exercise frequency. The heatmap shows the percentage distribution across matched caregiver and DS exercise categories, indicating a positive association between caregiver and DS family member physical activity. (**D**) Reported exercise frequency of individuals with DS stratified by age group, showing a decline in more frequent exercise with increasing age. (**E**) Reported exercise frequency of individuals with DS stratified by sex and caregiver-perceived obesity status. Bars show the number of respondents in each exercise-frequency category, with colors indicating caregiver-perceived obesity status. DS, Down syndrome. Percentages are calculated within each panel-specific subgroup. Numbers in parentheses indicate respondent counts. Labels omitted from the figure for readability; (**D**); (1–15/Monthly; n = 6 (3.0%); 16–30/Monthly; n = 8 (3.1%); 31–45/Monthly; n = 12 (5.2%); 46–50/Monthly; n = 2 (5.6%); 46–50/Daily; n = 2 (5.6%); 51+/4–6; n = 1 (3.7%); 51+/Daily; n = 1 (3.7%)); (**E**); (Male/Never/Probably not; n = 4 (10.5%); Male/Never/Don’t know; n = 2 (5.3%); Male/Never/Probably; n = 3 (7.9%); Male/1–3/Don’t know; n = 4 (1.8%); Male/4–6/Don’t know; n = 2 (2.4%); Male/4–6/Definitely; n = 8 (9.8%); Male/Daily/Probably not; n = 8 (20.0%); Male/Daily/Don’t know; n = 3 (7.5%); Male/Daily/Probably; n = 2 (5.0%); Male/Daily/Definitely; n = 2 (5.0%); Female/Never/Probably not; n = 5 (12.8%); Female/Never/Don’t know; n = 2 (5.1%); Female/4–6/Don’t know; n = 4 (5.2%); Female/4–6/Definitely; n = 8 (10.4%); Female/Daily/Probably not; n = 2 (8.3%); Female/Daily/Don’t know; n = 1 (4.2%); Female/Daily/Probably; n = 2 (8.3%); Female/Daily/Definitely; n = 4 (16.7%)).

We first examined exercise frequency among individuals with DS (DS physical activity, five categories; DS-PA_5_). Most were reported to exercise 1–3 times per week (55% of male, 56% of female), followed by 4–6 times per week (21% for both sexes). Overall patterns were broadly similar between males and females, although a higher proportion of males were reported to exercise daily (Figure 1A).

Caregiver exercise patterns were also investigated (Caregiver Exercise, five categories; CG-EX_5_). Most caregivers reported engaging in PA, with 45% exercising 2–3 days per week, and 23% exercising 4–5 days per week (Figure 1B). Smaller proportions reported once a week (18%) or no exercise at all (7%). Information on caregiver sex was not available and could therefore not be analyzed.

Spearman correlation analysis indicated a strong positive association between caregiver exercise frequency and that of their family members with DS (Spearman’s ρ = 0.521, *p* < 0.001; Figure 1C). When stratified by age, exercise frequency declined steadily across age groups (Figure 1D). Analysis by sex and perceived obesity revealed that among individuals exercising 1–3 times per week, a greater proportion of males than females were perceived by caregivers as “Definitely not” or “Probably not” obese (Figure 1E).

To examine whether the association between caregiver and DS physical activity persisted after adjustment for demographic characteristics, we fitted a binary logistic regression model with DS PA status as the outcome (DS-PA2; active vs. inactive) and caregiver PA level as the predictor (CG-EX3; low, moderate, high), adjusted for sex, age group, and region (n = 751; Table A1). Compared with caregivers in the low exercise group, caregivers in the moderate exercise group had thirteen-fold higher odds of having a family member with DS classified as active (aOR = 13.03, 95% CI: 7.40–24.06, *p* < 0.001), while those in the high exercise frequency group had more than seven-fold higher odds (aOR = 7.31, 95% CI: 4.16–13.44, *p* < 0.001). In contrast, the odds of being active decreased substantially with age, particularly among individuals aged 31–45 years (aOR = 0.4, 95% CI: 0.21–0.76, *p* = 0.006) and >46 years (aOR = 0.12, 95% CI: 0.05–0.25, *p* < 0.001), relative to the youngest age group. Sex (*p* = 0.28) and region (*p* = 0.78) were not significant predictors, and model fit was modest (McFadden R^2^ = 0.22).

### 3.2. Practices: Dietary Habits and Eating Patterns in Individuals with DS

**Figure 2:**
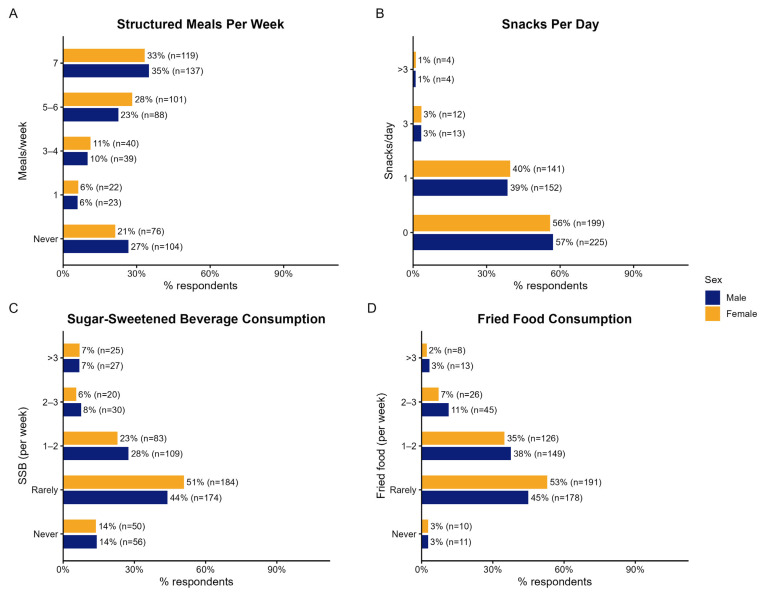
Structured meals and eating habits. (**A**). Frequency of structured meals per week stratified by sex. (**B**). Number of snacks consumed per day stratified by sex. (**C**). Frequency of sugar-sweetened beverage consumption per week, stratified by sex. (**D**). Frequency of fried food consumption per week, stratified by sex. Percentages are calculated within each sex group. Numbers in parentheses indicate respondent counts. Abbreviations: SSB, sugar-sweetened beverage.

We next examined dietary structure and selected food consumption patterns in individuals with DS. Most caregivers reported that their family members with DS followed regular structured meal patterns, defined as having three major meals and two minor meals per day. Structured meals were reported every day by 35% of males and 33% of females, and 5–6 days per week by 23% of males and 28% of females (Figure 2A). However, 6% of respondents reported structured meals only once per week. Daily snack intake was generally low. Most caregivers reported that their family member with DS consumed no daily snacks (57% of males and 56% of females), while approximately 40% reported one snack per day, with similar patterns across sex (Figure 2B).

SSBs and fried food consumption was also broadly similar between males and females. Most caregivers reported that SSBs were consumed “rarely” (44% of males and 51% of females), while 28% of males and 23% of females consumed SSBs 1–2 times per week (Figure 2C). Fried foods were also consumed “rarely” (45% of males and 53% of females), although more than one third of respondents reported fried food intake 1–2 times per week (38% of males and 35% of females; Figure 2D).

**Figure 3:**
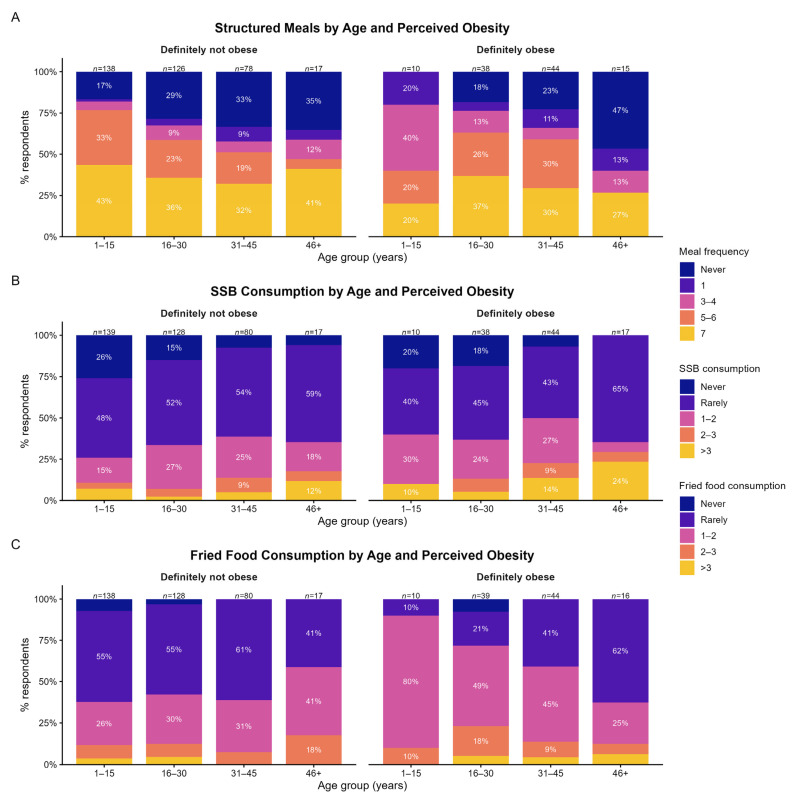
Dietary patterns by age group and caregiver-perceived obesity status in individuals with Down syndrome. (**A**) Frequency of structured meals per week, stratified by age group and caregiver-perceived obesity status. (**B**) Frequency of sugar-sweetened beverage consumption per week, stratified by age group and caregiver-perceived obesity status. (**C**) Frequency of fried food consumption per week, stratified by age group and caregiver-perceived obesity status. Only individuals perceived by caregivers as “Definitely not obese” or “Definitely obese” are shown. Percentages are calculated within each age and perceived-obesity subgroup. Numbers above bars indicate subgroup sample sizes. Abbreviations: SSB, sugar-sweetened beverage. Labels omitted from the figure for readability; (**A**); (Definitely not obese/16–30/1; n = 5 (4.0%); Definitely not obese/1–15/1; n = 2 (1.4%); Definitely not obese/1–15/3–4; n = 7 (5.1%); Definitely not obese/31–45/3–4; n = 5 (6.4%); Definitely not obese/46+/1; n = 1 (5.9%); Definitely not obese/46+/5–6; n = 1 (5.9%); Definitely obese/16–30/1; n = 2 (5.3%); Definitely obese/31–45/3–4; n = 3 (6.8%)); (**B**); (Definitely not obese/16–30/2–3; n = 6 (4.7%); Definitely not obese/16–30/>3; n = 3 (2.3%); Definitely not obese/1–15/2–3; n = 5 (3.6%); Definitely not obese/1–15/>3; n = 10 (7.2%); Definitely not obese/31–45/>3; n = 4 (5.0%); Definitely not obese/31–45/Never; n = 6 (7.5%); Definitely not obese/46+/2–3; n = 1 (5.9%); Definitely not obese/46+/Never; n = 1 (5.9%); Definitely obese/16–30/2–3; n = 3 (7.9%); Definitely obese/16–30/>3; n = 2 (5.3%); Definitely obese/31–45/Never; n = 3 (6.8%); Definitely obese/46+/1–2; n = 1 (5.9%); Definitely obese/46+/2–3; n = 1 (5.9%)); (**C**); (Definitely not obese/16–30/2–3; n = 10 (7.8%); Definitely not obese/16–30/>3; n = 6 (4.7%); Definitely not obese/16–30/Never; n = 4 (3.1%); Definitely not obese/1–15/2–3; n = 11 (8.0%); Definitely not obese/1–15/>3; n = 5 (3.6%); Definitely not obese/1–15/Never; n = 10 (7.2%); Definitely not obese/31–45/2–3; n = 6 (7.5%); Definitely obese/16–30/>3; n = 2 (5.1%); Definitely obese/16–30/Never; n = 3 (7.7%); Definitely obese/31–45/>3; n = 2 (4.5%); Definitely obese/46+/2–3; n = 1 (6.2%); Definitely obese/46+/>3; n = 1 (6.2%)).

Dietary behavior was further examined by age and perceived obesity (Figure 3) using Age_4. Individuals perceived as “Definitely not” obese reported more frequent structured meals than those perceived as “Definitely” obese, with 41% versus 27% reporting structured meals seven days per week, respectively (Figure 3A). SSB consumption was relatively stable across age groups among individuals perceived as “Definitely not” obese. In contrast, among those perceived as “Definitely” obese, the proportion reporting that they “Never” consumed SSBs decreased with age (Figure 3B). Reported fried food intake tended to decrease with age among individuals perceived as “Definitely not” obese but increased among those perceived as “Definitely” obese (Figure 3C). These age-related patterns should be interpreted cautiously because older participants represented a small proportion of the cohort, with approximately 9% aged ≥46 years.

To examine whether dietary behaviors were associated with perceived obesity, we fitted a binary logistic regression model with perceived obesity as the outcome (Yes = 321, No = 485, Don’t Know = excluded). SSB consumption was not a significant predictor at either moderate (aOR = 1.35, 95% CI 0.90–2.02) or high levels (aOR = 1.14, 95% CI: 0.67–1.92, *p* = 0.0632), relative to low consumption (Table A1). In contrast, moderate fried food consumption, defined as 1–2 times per week, was significantly associated with more than twice the odds of perceived obesity compared with low intake (aOR = 2.27, 95% CI: 1.56–3.32, *p* ≤ 0.001). High fried food consumption did not reach statistical significance (aOR = 1.61, 95% CI: 0.91–2.81, *p* = 0.096). Consuming two or more snacks per day was associated with four-fold higher odds of perceived obesity compared with consuming zero or one snack per day (aOR = 4.00, 95% CI: 1.78–9.22, *p* < 0.001). Region was not a significant predictor (*p* = 0.651), and model fit was modest (McFadden R^2^ = 0.12).

**Figure 4:**
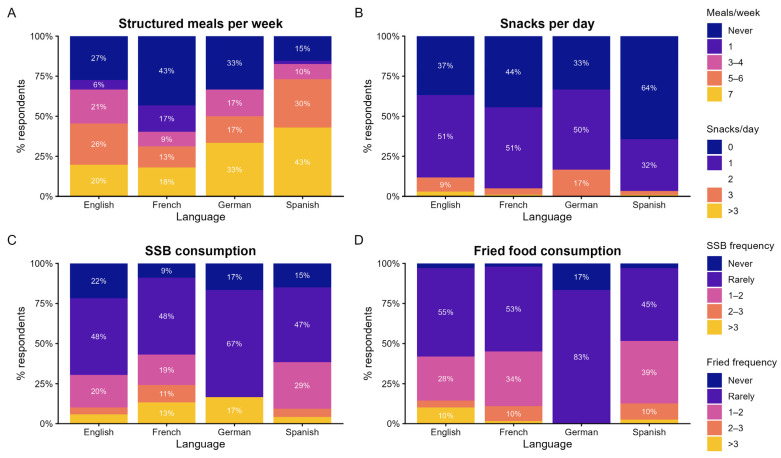
Dietary patterns by survey language group in individuals with Down syndrome. (**A**) Frequency of structured meals per week by language group. (**B**) Number of snacks consumed per day by language group. (**C**) Frequency of sugar-sweetened beverage consumption per week by language group. (**D**) Frequency of fried food consumption per week by language group. Percentages are calculated within each language group. German-speaking respondents are displayed descriptively but were excluded from formal comparative analyses because of the small sample size. Abbreviation: SSB, sugar-sweetened beverage. Labels omitted from the figure for readability; (**A**); (Spanish/1; n = 9 (1.9%); (**B**); (English/>3; n = 2 (2.9%); French/3; n = 8 (4.0%); French/>3; n = 2 (1.0%); Spanish/3; n = 12 (2.5%); Spanish/>3; n = 4 (0.8%)); (**C**); (English/2–3; n = 3 (4.3%); English/>3; n = 4 (5.8%); Spanish/2–3; n = 25 (5.2%); Spanish/>3; n = 20 (4.1%)); (**D**); (English/Never; n = 2 (2.9%); English/2–3; n = 3 (4.3%); French/Never; n = 4 (2.0%); French/>3; n = 3 (1.5%); Spanish/Never; n = 14 (2.9%); Spanish/>3; n = 12 (2.5%)).

Given that perceived obesity was similarly distributed across language groups in the companion paper (nutrients-4216283), we also explored dietary differences by language group. German-speaking respondents are shown in Figure 4 but were excluded from the formal analysis because of the small sample size. When stratified by language group, dietary patterns differed across groups (Figure 4A–D). Spanish-speaking respondents reported the most structured dietary habits with 43% reporting structured meals every day, and the lowest snack frequency, and 64% reporting no daily snacks. English-speaking respondents reported less frequent daily structured meals and higher snack intake, with only 37% reporting no daily snacks, 9% reporting three snacks per day, and 10% reporting fried food consumption more than three times per week. French-speaking respondents reported the lowest frequency of structured meals and the highest SSB consumption, with only 18% reporting structured meals every day and 14% reporting SSB consumption more than three times per week. These differences may reflect cultural variation in meal structure and dietary practices, although they should be interpreted cautiously given the descriptive nature of these analyses.

### 3.3. Attitudes: Interventional Beliefs Among Caregivers

**Figure 5:**
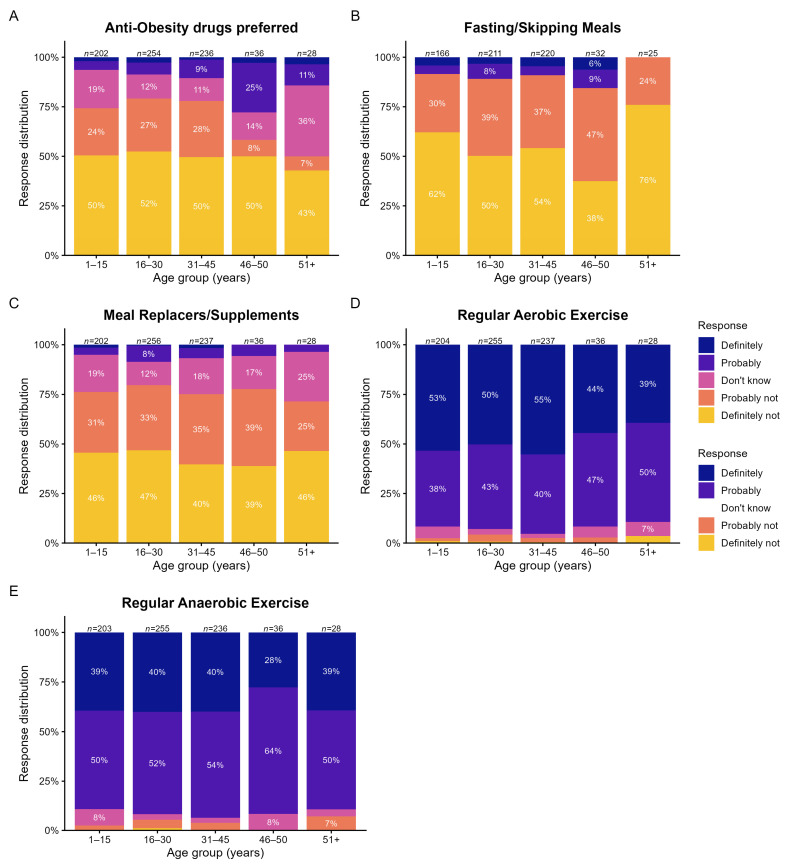
Caregiver beliefs regarding weight-loss intervention strategies for individuals with Down syndrome. Stacked bar charts show caregiver responses by age group for whether each strategy should be preferred for weight loss in their family member with DS: (**A**) anti-obesity drugs, (**B**) fasting or skipping meals, (**C**) meal replacers or supplements, (**D**) regular aerobic exercise, and (**E**) regular anaerobic exercise. Percentages are calculated within each age group. Numbers above bars indicate subgroup sample sizes. Abbreviation: DS, Down syndrome. Labels omitted for readability; (**A**); (1–15/Definitely; n = 4 (2.0%); 1–15/Probably; n = 9 (4.5%); 16–30/Definitely; n = 7 (2.8%); 16–30/Probably; n = 15 (5.9%); 31–45/Definitely; n = 3 (1.3%); 46–50/Definitely; n = 1 (2.8%); 51+/Definitely; n = 1 (3.6%)); (**B**); (1–15/Definitely; n = 7 (4.2%); 1–15/Probably; n = 7 (4.2%); 16–30/Definitely; n = 7 (3.3%); 31–45/Definitely; n = 10 (4.5%); 31–45/Probably; n = 10 (4.5%)); (**C**); (1–15/Definitely; n = 3 (1.5%); 1–15/Probably; n = 7 (3.5%); 16–30/Definitely; n = 1 (0.4%); 31–45/Definitely; n = 4 (1.7%); 31–45/Probably; n = 12 (5.1%); 46–50/Probably; n = 2 (5.6%); 51+/Probably; n = 1 (3.6%)); (**D**); (1–15/Don’t know; n = 12 (5.9%); 1–15/Probably not; n = 3 (1.5%); 1–15/Definitely not; n = 2 (1.0%); 16–30/Don’t know; n = 7 (2.7%); 16–30/Probably not; n = 9 (3.5%); 16–30/Definitely not; n = 2 (0.8%); 31–45/Don’t know; n = 5 (2.1%); 31–45/Probably not; n = 5 (2.1%); 31–45/Definitely not; n = 1 (0.4%); 46–50/Don’t know; n = 2 (5.6%); 46–50/Probably not; n = 1 (2.8%); 51+/Definitely not; n = 1 (3.6%)); (**E**); (1–15/Probably not; n = 5 (2.5%); 16–30/Don’t know; n = 7 (2.7%); 16–30/Probably not; n = 11 (4.3%); 16–30/Definitely not; n = 3 (1.2%); 31–45/Don’t know; n = 6 (2.5%); 31–45/Probably not; n = 9 (3.8%); 51+/Don’t know; n = 1 (3.6%)).

Caregivers were asked about their beliefs regarding different weight-loss intervention strategies for their family members with DS (Figure 5A–E). Across all age groups, most caregivers did not endorse anti-obesity drugs, fasting/skipping meals, or meal replacers/supplements as preferred interventions. Endorsement of anti-obesity drugs varied by age group, with the highest proportion observed among caregivers of individuals aged 46–50 years, of whom 25% selected “Probably” (Figure 5A). Greater uncertainty was observed among caregivers of individuals aged >51 years, with 36% selecting “Don’t know.” In contrast, regular aerobic and anaerobic exercise were the most frequently endorsed weight-loss strategies across all age groups, with most caregivers selecting either “Definitely” or “Probably” (Figure 5D,E).

**Figure 6:**
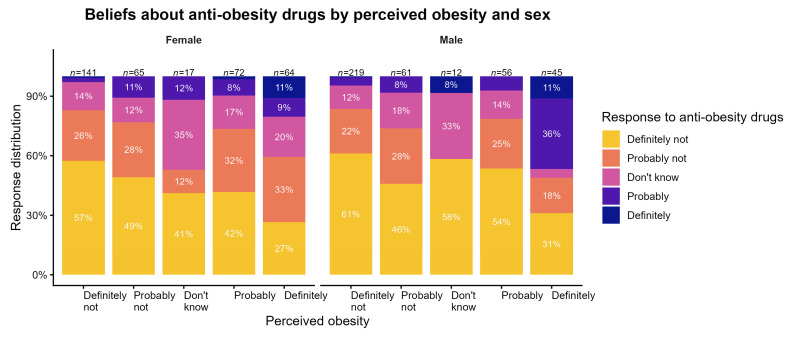
Caregiver beliefs about anti-obesity drugs by perceived obesity status and sex. Stacked bar charts show caregiver responses on whether anti-obesity drugs should be preferred as a weight-loss strategy, stratified by caregiver-perceived obesity status and sex of the family member with DS. Percentages are calculated within each perceived-obesity and sex subgroup. Numbers above bars indicate subgroup sample sizes. Abbreviation: DS, Down syndrome. Labels omitted from the figure for readability: (Female/Definitely not/Definitely; n = 1 (0.7%); Female/Definitely not/Probably; n = 3 (2.1%); Female/Probably/Definitely; n = 1 (1.4%); Male/Definitely not/Definitely; n = 1 (0.5%); Male/Definitely not/Probably; n = 9 (4.1%); Male/Probably/Probably; n = 4 (7.1%); Male/Definitely/Don’t know; n = 2 (4.4%)).

When responses were stratified by caregiver-perceived obesity status and sex, endorsement of anti-obesity drugs was higher when the family member with DS was perceived as “Definitely” obese (Figure 6). Among individuals perceived as “Definitely” obese, a higher proportion of caregivers endorsed anti-obesity drugs for males than for females, with 47% versus 19% selecting “Definitely” or “Probably,” respectively.

To formally assess these patterns, binary logistic regression models were fitted. For anti-obesity drug endorsement, perceived obesity was associated with more than four-fold higher odds of endorsing anti-obesity medication (aOR = 4.21, 95% CI: 2.44–7.39, *p* < 0.001). Older age was also a significant predictor, with caregivers of individuals aged >46 years having higher odds of endorsement than those caring for individuals aged 1–15 years (aOR = 2.74, 95% CI: 1.09–6.87, *p* = 0.031). Sex and younger age groups were not significant predictors, and model fit was modest (McFadden R^2^ = 0.108; Table A1). For meal replacers/supplements, perceived obesity was also associated with higher odds of endorsement (aOR = 2.26, 95% CI: 1.22–4.21, *p* = 0.010), with no significant associations for sex, age, or region (McFadden R^2^ = 0.035). For fasting/skipping meals, no predictor reached statistical significance (McFadden R^2^ = 0.010).

### 3.4. Attitudes: Caregiver Confidence in DS Family Member Engaging in Healthier Behaviors

**Figure 7:**
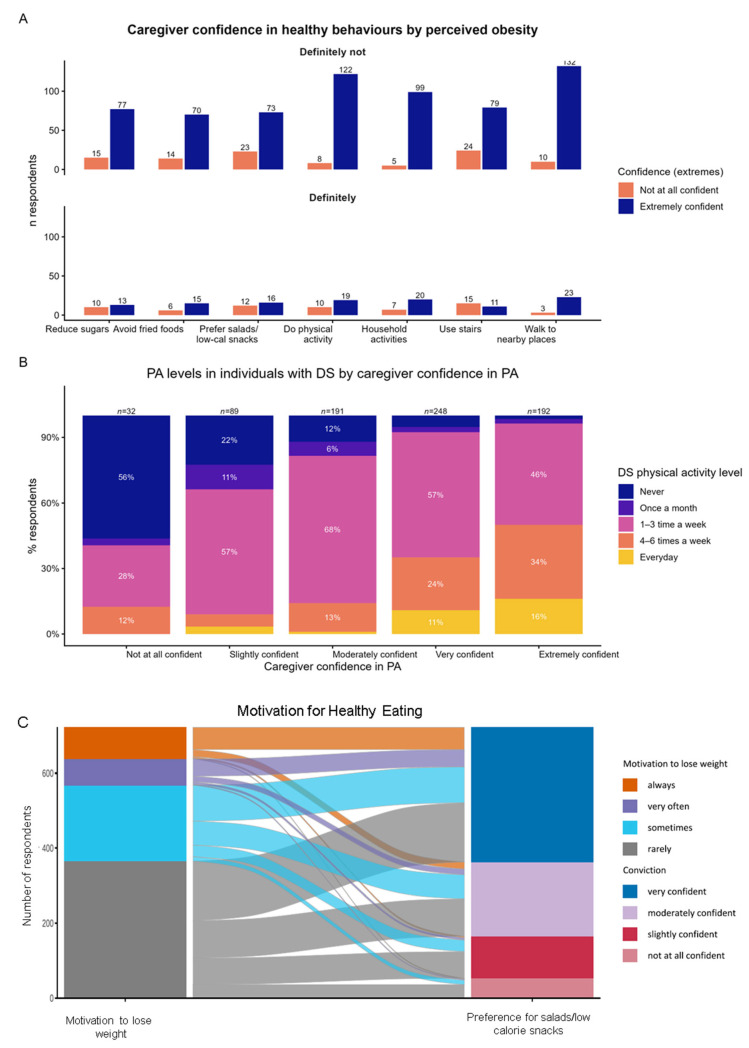
Caregiver confidence in healthier behaviors among individuals with Down syndrome. (**A**) Caregiver confidence that their family member with DS would engage in selected healthier behaviors, stratified by caregiver-perceived obesity status. Bars show the number of caregivers selecting the two extreme confidence categories: “Not at all confident” and “Extremely confident.” (**B**) Reported physical activity frequency of individuals with DS according to caregiver confidence that their family member would engage in physical activity. Percentages are calculated within each caregiver-confidence category. (**C**) Alluvial plot showing the relationship between perceived motivation to lose weight and caregiver confidence that the family member with DS would choose lower-calorie snacks over sweet, fried, or refined foods. Responses for “Rarely” and “Never” motivated to lose weight were grouped, and responses for “Very confident” and “Extremely confident” were grouped for confidence in healthier dietary choices. Abbreviations: DS, Down syndrome; PA, physical activity. Labels omitted from the figure for readability; (**B**); (Not at all confident/Once a month; n = 1 (3.1%); Slightly confident/4–6 times a week; n = 5 (5.6%); Slightly confident/Everyday; n = 3 (3.4%); Moderately confident/Everyday; n = 2 (1.0%); Very confident/Never; n = 13 (5.2%); Very confident/Once a month; n = 6 (2.4%); Extremely confident/Never; n = 3 (1.6%); Extremely confident/Once a month; n = 4 (2.1%)).

Caregiver confidence in their family member’s ability to engage in healthier behaviors, including physical activity, avoiding fried foods, reducing sugar intake, using stairs, and walking short distances, differed by caregiver-reported perceived obesity status (Figure 7A). Caregivers who perceived their family member as “Definitely not” obese reported higher confidence across all behaviors. For example, “Extremely confident” responses were most frequent for walking to nearby places (n = 132), engaging in physical activity (n = 122), and household activities (n = 99). In contrast, among caregivers who perceived their family member as “Definitely” obese, “Extremely confident” responses were less frequent across behaviors, ranging from 11 to 23 responses.

We next examined caregiver confidence in their family member’s engagement in physical activity in relation to reported sport frequency (Figure 7B). Among caregivers who were “Extremely confident,” 50% of individuals with DS exercised 4–6 times per week or every day. Conversely, among caregivers who were “Not at all confident,” 56% of individuals with DS were reported to never exercise.

An ordinal cumulative link model was fitted with DS physical activity frequency as the outcome and caregiver confidence as the primary predictor, adjusted for demographic variables (n = 750). Increasing caregiver confidence was associated with greater odds of the family member with DS being in a higher physical activity category. Compared with caregivers who were “Not at all confident,” the adjusted odds ratios were 2.66 for “Slightly confident” (95% CI: 1.16–6.23, *p* = 0.022), 4.95 for “Moderately confident” (95% CI: 2.25–11.12, *p* < 0.001), 14.68 for “Very confident” (95% CI: 6.59–33.40, *p* < 0.001), and 25.63 for “Extremely confident” (95% CI: 11.30–59.36, *p* < 0.001; Table A1). Older age was associated with lower physical activity, particularly among individuals aged 31–45 years (aOR = 0.56, 95% CI: 0.38–0.82, *p* = 0.003) and ≥46 years (aOR = 0.39, 95% CI: 0.21–0.70, *p* = 0.002), compared with the youngest age group. Individuals from Other Europe had lower odds of being in a higher physical activity category than those from Spain (aOR = 0.65, 95% CI: 0.48–0.88, *p* = 0.006). However, the proportional odds assumption was violated for region (*p* = 0.001), so this regional effect should be interpreted with caution. Sex was not a significant predictor (*p* = 0.677).

Finally, we explored the relationship between perceived motivation to lose weight and caregiver confidence in healthier dietary choices (Figure 7C). Most caregivers reported that their family member with DS was “Rarely” motivated to lose weight. However, many of these caregivers also reported being “Very” or “Extremely confident” that their family member would choose lower-calorie snacks over sweet, fried, or refined foods. This suggests that confidence in specific healthier behaviors may not necessarily depend on perceived motivation to lose weight.

## 4. Discussion

In this study, we examined PA, dietary patterns, caregiver confidence, and perceptions of weight-loss interventions in a large European caregiver survey of individuals with DS. We found that PA levels in individuals with DS were positively associated with caregiver PA, while PA declined with age. Dietary patterns were heterogeneous, and fried food consumption and higher snack frequency were associated with caregiver-perceived obesity. Caregivers strongly favored exercise as a weight-loss strategy, whereas anti-obesity drugs, fasting/skipping meals, and meal replacers were less frequently endorsed. Caregiver confidence in the ability of individuals with DS to engage in healthier behaviors also differed by perceived obesity status and was strongly associated with reported PA levels.

### 4.1. The Role of Sex, Age, and Caregivers in Shaping Exercise Habits in DS

Regular PA is associated with improved cardiometabolic health and functional capacity [16]. However, individuals with DS often do not meet the recommended activity levels [16,17,18]. In our survey, most individuals with DS exercised 1–3 times per week, while daily exercise was reported more often in males than females. Among those exercising 1–3 times per week, males were more frequently perceived as “Definitely not” or “Probably not” obese compared with females. This suggests that PA frequency alone may not fully capture relevant differences in exercise intensity, body composition, or fat distribution.

A key finding was the strong association between caregiver and DS family member activity levels. Individuals with DS whose caregivers reported moderate or high PA had substantially higher odds of being classified as active, even after adjustment for demographic characteristics. This alignment may reflect shared household routines and is consistent with previous evidence that caregiver and family engagement influence healthy behaviors in individuals with DS and other intellectual disabilities [19].

PA frequency also declined with age, consistent with previous studies reporting low PA and high sedentary behavior in adults with DS [20,21]. This decline may reflect changes in functional capacity, daily structure, opportunities for PA, or DS-specific physiological features such as lower resting metabolism, higher adiposity, and lower aerobic capacity. As these factors were not directly assessed, they should be interpreted cautiously. Nevertheless, the association between caregiver and family member activity suggests that family-based PA promotion may be a relevant intervention target.

### 4.2. Dietary Patterns and Food Structure in Individuals with DS

Understanding dietary patterns in individuals with DS is important because regular meal patterns have been associated with better nutrient intake and diet quality [22]. In this cohort, dietary patterns were heterogeneous, with approximately one third of caregivers reporting structured meals every day. Because meal structure was assessed as frequency per week rather than dietary composition, these findings reflect the regularity of eating patterns rather than nutritional quality. Structured meal frequency declined with age, although older participants were underrepresented, suggesting that the cohort may mainly reflect individuals with DS still living at home.

Frequent consumption of SSBs and fried foods was uncommon, in line with previous studies reporting relatively low regular SSB consumption in individuals with DS, particularly in younger populations [2,23]. However, dietary patterns varied by perceived obesity status. In adjusted analyses, moderate fried food consumption and higher snack frequency were associated with more than twofold and fourfold higher odds of perceived obesity, respectively, whereas SSB consumption was not a significant predictor. Given the cross-sectional design and caregiver-reported nature of the data, causal relationships cannot be established.

Dietary patterns also differed across language groups, potentially reflecting cultural differences in meal structure, food choices, and snacking behaviors [24]. Spanish-speaking respondents reported more structured meals and lower snack frequency than French- or English-speaking respondents, consistent with dietary patterns often associated with Mediterranean eating habits [25]. In fact, within the GO-DS21 study, we have identified notable cross-country differences in the dietary patterns of individuals with DS [22]. However, perceived obesity was similarly distributed across countries in the companion study, suggesting that structured meal frequency alone does not explain obesity status. These findings should be interpreted cautiously because dietary data were caregiver-reported and did not include quantitative measures of intake, nutritional quality, or energy balance.

### 4.3. Confidence, Interventional Beliefs and Barriers

Weight-management guidance for individuals with DS emphasizes tailored, multicomponent approaches combining dietary modification, PA, and behavioral strategies [26]. In our cohort, caregiver confidence was strongly associated with reported PA in individuals with DS. Those whose caregivers were extremely confident in their engagement in PA had more than 25-fold higher odds of being in a higher PA category compared with those whose caregivers were not confident. However, because the proportional odds assumption was violated for region, regional comparisons should be interpreted cautiously.

Although causality cannot be inferred, these findings suggest that caregiver confidence may be closely linked to PA participation. Confidence was lower when individuals were perceived as obese, which may reflect caregivers’ experience of functional limitations or previous difficulties with lifestyle change. DS-associated characteristics such as reduced muscle mass, higher adiposity, and lower metabolic rate may also influence exercise tolerance. Interestingly, caregivers often reported low motivation for weight loss among individuals with DS while expressing confidence in their ability to adopt specific healthier behaviors, such as choosing lower-calorie snacks. This suggests that individual healthy behaviors may be more feasible when integrated into daily routines rather than framed only around weight loss.

Caregivers strongly endorsed aerobic and anaerobic exercise as preferred weight-loss strategies, whereas support for anti-obesity drugs, fasting/skipping meals, and meal replacers was low. Support for pharmacological approaches was mainly associated with perceived obesity, while sex was not significant after adjustment. Although anti-obesity medications have demonstrated efficacy in the general population [13], confidence in individuals with DS remains limited. Caregiver hesitation may therefore reflect uncertainty about safety, long-term effects, or suitability. Overall, these findings support the value of caregiver-inclusive interventions that promote shared PA and practical lifestyle strategies.

## 5. Conclusions

This study highlights the association between caregiver behaviors, caregiver confidence, and lifestyle patterns in individuals with DS. Caregiver PA was strongly associated with PA levels in family members with DS, suggesting that physical activity may cluster within households and that family-based approaches could support healthier routines.

Dietary findings showed that fried food consumption and higher snack frequency were independently associated with perceived obesity, whereas SSB consumption was not. These behaviors may represent potential targets for tailored dietary guidance, although conclusions are limited by caregiver-reported data and the absence of quantitative dietary intake measures.

Caregivers strongly favored exercise as a weight-loss strategy, while pharmacological approaches were mainly considered when obesity was perceived. Future interventions to reduce obesity risk in individuals with DS may benefit from involving both individuals with DS and their caregivers, while considering the broader social, functional, and environmental factors that shape lifestyle behaviors.

## Data Availability

The anonymized survey dataset supporting the findings of this study is available in the Zenodo repository: https://doi.org/10.5281/zenodo.18863735. The survey is available at https://seatable-ics.igbmc.fr/dtable/forms/dda98c1a-cca0-44fd-b77f-5a098217acd4/ (accessed on 26 February 2026).

## Abbreviations

The following abbreviations are used in this manuscript:

DS	Down syndrome
SSB	Sugar-sweetened beverage
WHO	World Health Organization
PA	Physical activity
NCD	Non-communicable disease

## Appendix A

**Table A1:** Adjusted odds ratio (aOR) and 95% confidence intervals from regression analyses examining associations between caregiver characteristics, dietary behaviors, and physical activity outcomes in individuals with Down syndrome.

Predictor	aOR	95% CI	*p*
DS PA frequency~Caregiver Confidence (Ordinal CLM, n = 750)			
Caregiver confidence (ref: Not at all confident)			
Slightly confident	2.66	1.16–6.23	0.022
Moderately confident	4.95	2.25–11.12	<0.001
Very confident	14.68	6.59–33.40	<0.001
Extremely confident	25.63	11.30–59.36	<0.001
Covariates			
Sex (Female vs. Male)	0.94	0.71–1.25	0.677
Age: 16–30 vs. 1–15 years	0.87	0.61–1.26	0.463
Age: 31–45 vs. 1–15 years	0.56	0.38–0.82	0.003
Age: 46+ vs. 1–15 years	0.39	0.21–0.70	0.002
Region (Other Europe vs. Spain)	0.65	0.48–0.88	0.006
Model 2: DS PA Active/Inactive~Caregiver Exercise (Binary Logistic, n = 751)			
Caregiver exercise (ref: Low)			
Moderate (2–3 days/week)	13.03	7.40–24.06	<0.001
High (4–7 days/week)	7.31	4.16–13.44	<0.001
Covariates			
Sex (Female vs. Male)	0.78	0.49–1.23	0.28
Age: 16–30 vs. 1–15 years	0.89	0.45–1.74	0.74
Age: 31–45 vs. 1–15 years	0.4	0.21–0.76	0.006
Age: 46+ vs. 1–15 years	0.12	0.05–0.25	<0.001
Region (Other Europe vs. Spain)	0.94	0.58–1.52	0.785
Model 3: Perceived Obesity~Dietary Behaviors (Binary Logistic, n = 716)			
SSB consumption (ref: Low)			
Moderate (1–2×/week)	1.35	0.90–2.02	0.144
High (≥3×/week)	1.14	0.67–1.92	0.632
Fried food consumption (ref: Low)			
Moderate (1–2×/week)	2.27	1.56–3.32	<0.001
High (≥3×/week)	1.61	0.91–2.81	0.096
Snack frequency (≥2/day vs. 0–1/day)	4	1.78–9.22	<0.001
Covariates			
Sex (Female vs. Male)	2.2	1.57–3.12	<0.001
Age: 16–30 vs. 1–15 years	2.97	1.81–5.00	<0.001
Age: 31–45 vs. 1–15 years	4.74	2.89–7.96	<0.001
Age: 46+ vs. 1–15 years	7.42	3.74–14.99	<0.001
Region (Other Europe vs. Spain)	1.09	0.75–1.57	0.651
Interventional Belief Endorsement (Binary Logistic)			
Anti-obesity drugs (n = 623)			
Perceived obese (vs. not obese)	4.21	2.44–7.39	<0.001
Age: 46+ vs. 1–15 years	2.74	1.09–6.87	0.031
Sex (Female vs. Male)	0.73	0.43–1.23	0.24
Meal replacers (n = 615)			
Perceived obese (vs. not obese)	2.26	1.22–4.21	0.01
Sex (Female vs. Male)	0.87	0.48–1.58	0.654
Fasting/skipping meals (n = 626)			
Perceived obese (vs. not obese)	1.45	0.80–2.59	0.21
Sex (Female vs. Male)	0.92	0.53–1.60	0.77
